# MTBVAC induces superior antibody titers and IgG avidity compared to BCG vaccination in non-human primates

**DOI:** 10.1038/s41541-024-01009-5

**Published:** 2024-11-20

**Authors:** Marco Polo Peralta-Álvarez, Keya Downward, Andrew White, Hugo Redondo Azema, Laura Sibley, Charlotte Sarfas, Alexandra Morrison, Mike Dennis, Delia Diaz-Santana, Stephanie A. Harris, Shuailin Li, Eugenia Puentes, Nacho Aguilo, Carlos Martin, Sally Sharpe, Helen McShane, Rachel Tanner

**Affiliations:** 1 Jenner Institute, Nuffield Department of Medicine, University of Oxford, Oxford, UK; 2 Laboratorio Nacional de Vacunologia y Virus Tropicales, Departamento de Microbiologia, Escuela Nacional de Ciencias Biologicas, Instituto Politecnico Nacional, Ciudad de Mexico, Mexico; 3 UK Health Security Agency, Porton Down, Salisbury, UK; 4 École Normale Supérieure - PSL, Paris, France; 5 Department of Biology, University of Oxford, Oxford, UK; 6 Clinical Research Department y Research and Development Department, Biofabri, Grupo Zendal, O’Porriño, Pontevedra, Spain; 7 Faculty of Medicine, University of Zaragoza, Zaragoza, CIBERES, Instituto de Salud Carlos III, Madrid, Spain

**Keywords:** Live attenuated vaccines, Experimental models of disease

## Abstract

The only currently licensed vaccine against tuberculosis (TB), Bacille Calmette Guérin (BCG), is insufficient to control the epidemic. MTBVAC is a live attenuated strain of *Mycobacterium tuberculosis* (*M.tb*) and is one the most advanced TB vaccine candidates in the pipeline. It is more efficacious than BCG in preclinical models including non-human primates (NHPs), and has demonstrated safety and immunogenicity in human populations. To better understand the immune mechanisms underlying the superior efficacy conferred by MTBVAC, we characterized *M.tb*-specific antibody responses in NHPs vaccinated with either BCG or MTBVAC. MTBVAC vaccination induced higher titers of IgG, IgM and IgA, and higher avidity IgG compared with BCG vaccination. IgG avidity correlated with protection following *M.tb* challenge in the same animals, validating the association previously reported between this measure and protection in the context of intravenous BCG vaccination, suggesting that IgG avidity may represent a relevant marker or correlate of protection from TB.

## Introduction

Tuberculosis (TB) is the leading cause of death due to an infectious disease, with the World Health Organisation (WHO) reporting 1.3 million deaths in 2022^1,2^. The only available TB vaccine is Bacillus Calmette Guérin (BCG): a live-attenuated form of *Mycobacterium bovis* first administered to humans over a century ago. One of the major causes of BCG attenuation is the loss of the differential region RD1, which contains important antigens present in clinical *Mycobacterium tuberculosis* (*M.tb*) strains^3^. While BCG confers reliable protection against severe forms of TB in infancy, its efficacy against pulmonary disease is extremely variable in adolescents and adults and is particularly low in many TB endemic regions^4–6^. As pulmonary TB represents the principal source of bacillary spread, the impact of BCG vaccination on transmission of *M.tb* is minimal. A new and more effective TB vaccine that improves upon BCG is urgently needed.

Among various strategies aiming to improve on protection conferred by BCG against pulmonary TB is the use of live attenuated mutants of *M.tb* itself containing major antigens present in the RD1 region, based on the rationale that genetic proximity to human trophic *M.tb* may induce a more comprehensive and biologically relevant immune response^7–10^. One of the most advanced vaccine candidates in this arena is MTBVAC, a live attenuated *M.tb* strain that was developed by rational genetic modification including the deletion of two independent virulence genes, *phoP* and *fadD26*^11,12^. PhoP is involved in intracellular growth, immune evasion, and the secretion of the important immunogenic virulence factor Early Secretory Antigenic Target of 6-kDa of *M.tb* (ESAT-6)^13^. In addition, PhoP controls the synthesis of lipids such as sulfolipid, polyacyltrehaloses (PAT) and diacyltrehalose (DAT), which are major components of the mycobacterial cell wall and interfere with the immune system. FadD26 plays a critical role in the synthesis of complex lipids such as phthiocerol dimycocerosate (DIM) – a major virulence factor for *M.tb*. Thus, their independent deletions effectively attenuate the strain, making it safe for use in humans while still eliciting a strong immune response^12,14^. In fact, MTBVAC results in enhanced immunogenicity relative to the wild-type strain, which may be attributed to the higher secretion of immunogenic antigens such as Ag85 complex proteins and lipoarabinomannan (LAM)^15^. Recent metabolomic studies have also shown that MTBVAC has an increased production of phosphatidyl-inositol mannosides (PIMs), a structural component of the outer membrane of mycobacteria, compared to wild-type strains^16^.

A wide range of preclinical studies evaluating MTBVAC have indicated promising results. Safety, immunogenicity and efficacy have been demonstrated in several animal models including mice, guinea pigs and non-human primates (NHPs), with the immunogenic profile and levels of protection against *M.tb* challenge found to be comparable to, if not surpassing, those conferred by BCG^17–20^. The first phase I clinical trial of MTBVAC conducted at the Centre Hospitalier Universitaire Vaudois (Lausanne, Switzerland) enroled 36 healthy HIV-negative, QuantiFERON (QFT) negative, BCG-naïve volunteers aged between 18 and 45 years and showed that MTBVAC had a safety and immunogenic profile similar to that of BCG^21^. Polyfunctional T cells were induced in a dose-dependent manner with a trend for higher responses in the MTBVAC vaccinated group compared with BCG vaccination at the same dose^21^.

In a subsequent randomised, double-blind, controlled dose-escalation trial conducted in South Africa, infants received either BCG vaccination or a dose escalation of MTBVAC, with a safety arm in adults previously vaccinated at birth with BCG. The reactogenicity and safety of the highest dose of MTBVAC showed that MTBVAC was as safe as BCG^14^. MTBVAC induced a durable antigen-specific Th1 cytokine-expressing CD4^+^ T cell response in infants, which peaked 70 days after vaccination and was detectable for up to 12 months^14^. An ongoing Phase III efficacy trial aims to evaluate the safety, immunogenicity and efficacy of MTBVAC in HIV-uninfected infants born to HIV-infected and HIV-uninfected mothers in Sub-Saharan Africa (NCT04975178). This study will be critical for evaluating the potential use of MTBVAC in high TB incidence settings^19^.

Relatively little is known about the antibody responses induced by BCG or other TB vaccine candidates as few studies have measured this parameter^22^. While protection from TB has traditionally been considered to be mediated by cellular immunity, a growing body of literature suggests a role for antibodies^23^. In the context of BCG vaccination, an early study by de Vallière et al. reported significant induction of LAM-specific IgG following both primary and secondary BCG vaccination in healthy volunteers. Incubation with post-BCG vaccination serum significantly increased BCG internalization and growth restriction by macrophages in a mycobacterial growth inhibition assay in vitro; an effect that was reversible by pre-absorption of IgG^24^. In addition, in BCG-vaccinated South African infants, an association has been reported between levels of Ag85A-specific antibodies and a reduced risk of TB disease^25^. The M72/AS01_E_ vaccine candidate has been shown to be a potent inducer of *M.tb*-specific antibodies which were sustained throughout the 3-year follow-up period, although it remains to be determined whether these contribute to the ~50% efficacy observed against progression to TB disease in *M.tb*-infected individuals^26^.

Given the superior efficacy conferred by MTBVAC in preclinical studies, it is key to study the underlying immune mechanisms and inform a broader understanding of humoral correlates of protection from TB.

## Results

### Vaccination with MTBVAC induces superior *M.tb*-specific antibody titers compared with BCG

**Fig. 1:**
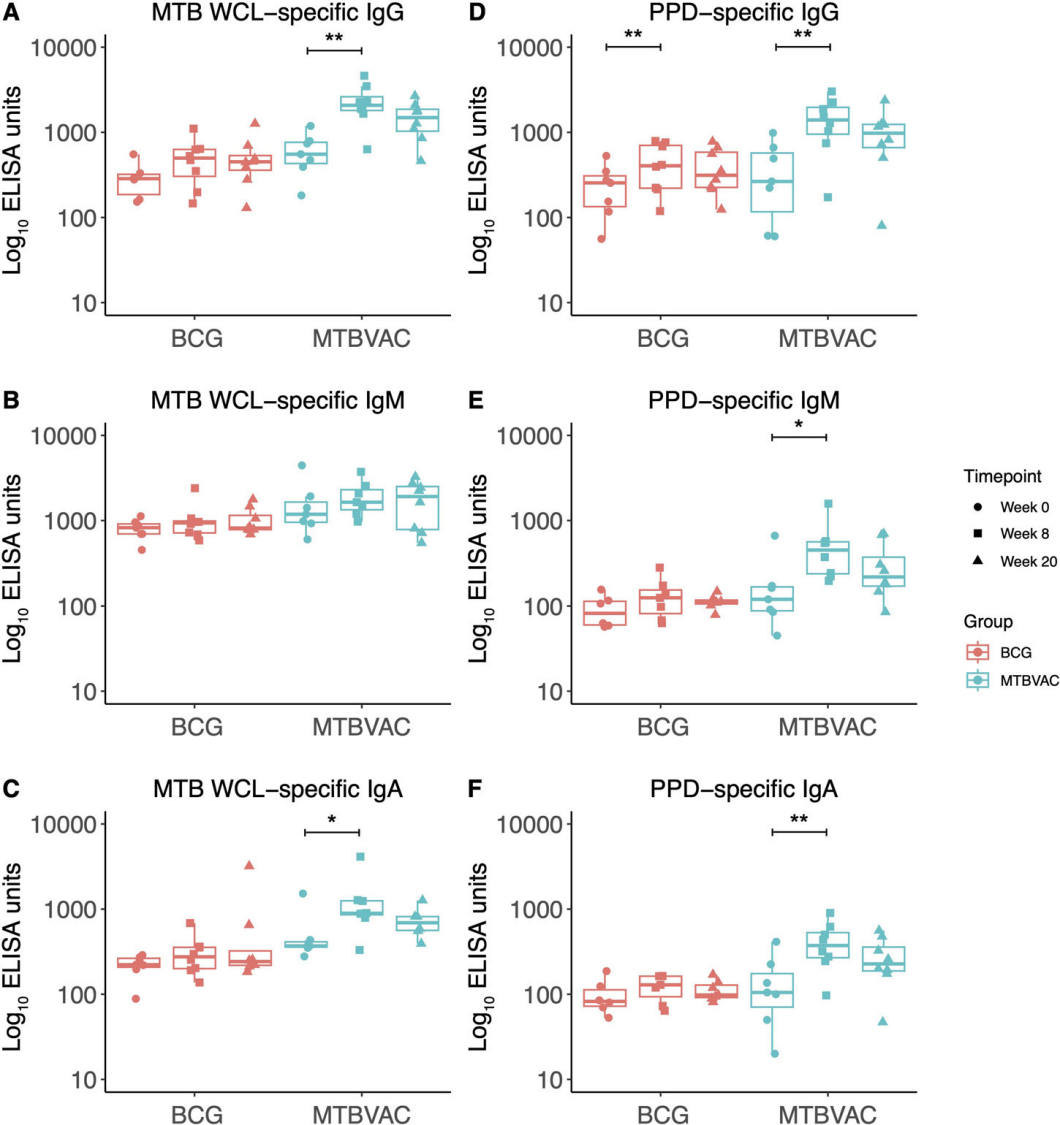
*M.tb*-specific antibody responses following BCG or MTBVAC vaccination. IgG (**A**), IgM (**B**) and IgA (**C**) responses to *M.tb* WCL in the sera of rhesus macaques collected at baseline, 8 and 20 weeks post-ID BCG vaccination (red, *n* = 6–8) or MTBVAC vaccination (cyan, *n* = 7–8). IgG (**D**), IgM (**E**) and IgA (**F**) responses to PPD in the sera of rhesus macaques collected at baseline, 8 and 20 weeks post-ID BCG vaccination (red, *n* = 6–8) or MTBVAC vaccination (cyan, *n* = 7–8). *p < 0.05, **p < 0.01. Bars represent the median and IQR.

Antibody responses to *M.tb* whole cell lysate (WCL) and purified protein derivative (PPD) were evaluated in serum collected from NHPs vaccinated with either BCG or MTBVAC by the intradermal (ID) route, at baseline and at 8 and 20 weeks post-vaccination. There was a significant increase in PPD-specific IgG at 8 weeks following BCG vaccination (*p* = 0.001), but no other differences in this group (Fig. 1A–F). There was a significant increase in *M.tb* WCL-specific IgG (*p* = 0.001, Fig. 1A) and IgA (*p* = 0.04, Fig. 1C), but not IgM, at 8 weeks post-MTBVAC vaccination. There was also a significant increase in PPD-specific IgG (*p* = 0.001, Fig. 1D), IgM (*p* = 0.02, Fig. 1E) and IgA (*p* = 0.007, Fig. 1F) at 8 weeks post-MTBVAC vaccination. For the MTBVAC vaccinated group, there was a correlation between PPD-specific and *M.tb* WCL-specific IgG and IgA (but not IgM) at week 8 (*r* = 0.93, *p* = 0.002 and *r* = 0.81, *p* = 0.02 respectively; Supplementary Fig. 1A–C), and a significant correlation between PPD-specific and *M.tb* WCL-specific IgG, IgM and IgA at week 20 (*r* = 0.98, *p* = 0.0004; *r* = 0.86, *p* = 0.01 and *r* = 1, *p* = 0.0004 respectively; Supplementary Fig. 1D–F). There were no significant correlations between responses to the two antigen mixtures for the BCG-vaccinated group.

PPD-specific ELISpot responses following BCG or MTBVAC vaccination in the same animals have been previously reported^20^. When both groups were combined, there was no association between frequency of PPD-specific IFN-γ secreting cells and measures of protection following *M.tb* challenge (Supplementary Fig. 2A). Similarly, there were no associations when each group was analysed separately (data not shown). To investigate the influence of cellular immunity on antibody responses, correlations between PPD-specific ELISpot responses and PPD-specific antibody titres were analysed. When both groups were combined, there was a significant association between frequency of PPD-specific IFN-γ secreting cells at 16 weeks post-vaccination and titres of PPD-specific IgG, IgA and IgM at week 8 (*r* = 0.75, *p* = 0.001; *r* = 0.52, *p* = 0.05 and *r* = 0.59, *p* = 0.02 respectively) and at week 20 post-vaccination (*r* = 0.68, *p* = 0.004; *r* = 0.64, *p* = 0.01 and *r* = 0.66, *p* = 0.008 respectively) (Supplementary Fig. 2B). This association held up in the MTBVAC (but not BCG) vaccinated group when analysed alone at week 8 (*r* = 0.76, *p* = 0.03; *r* = 0.86, *p* = 0.007 and *r* = 0.71, *p* = 0.047 respectively) and week 20 post-vaccination (*r* = 0.86, *p* = 0.007; *r* = 0.76, *p* = 0.03 and *r* = 0.79, *p* = 0.02 respectively) (data not shown).

ESAT-6 and CFP-10 are major immunogenic proteins that are encoded in the RD1 region and thought to be co-secreted^27^; they are present in MTBVAC but not BCG as the RD1 region is deleted in the latter. Due to the PhoP deletion in MTBVAC, ESAT-6 is expressed but not exported, while CFP-10 is expressed and exported. White et al. found an increase in CFP-10-specific but not ESAT-6-specific IFN-γ ELISpot responses following MTBVAC vaccination that was significantly higher than that following BCG vaccination^20^. We thus explored ESAT-6 and CFP-10 specific IgG responses but did not see any significant change in the response to either antigen following BCG or MTBVAC vaccination - although two animals showed an increase in CFP-10 response at 20 weeks post-MTBVAC (Supplementary Fig. 3A,B). Interestingly there was a significant inverse correlation between CFP-10 IgG response at 8 weeks post-MTBVAC and gross pathology score (*r* = −0.71, *p* = 0.047).

### IgG subclass analysis demonstrates superior IgG1 responses and IgG1/IgG3 ratio following MTBVAC vaccination

**Fig. 2:**
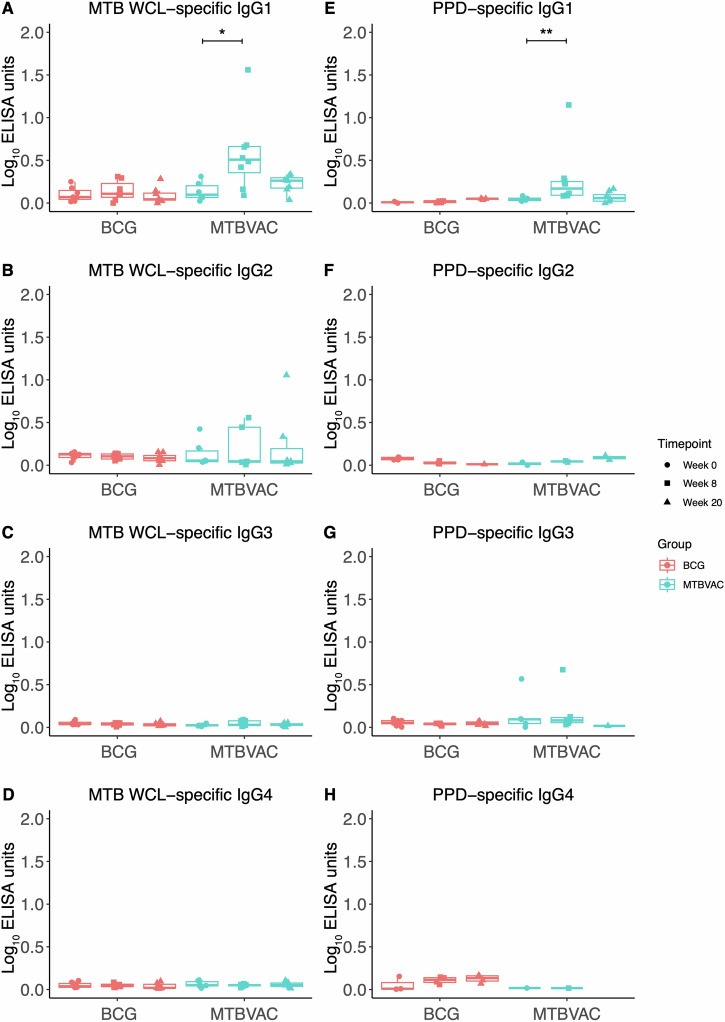
*M.tb*-specific IgG subclass responses following BCG or MTBVAC vaccination. IgG1 (**A**), IgG2 (**B**), IgG3 (**C**) and IgG4 (**D**) responses to *M.tb* WCL in the sera of rhesus macaques collected at baseline, 8 and 20 weeks post-ID BCG vaccination (red, *n* = 7–8) or MTBVAC vaccination (cyan, *n* = 6–8). IgG1 (**E**), IgG2 (**F**), IgG3 (**G**) and IgG4 (**H**) responses to PPD in the sera of rhesus macaques collected at baseline, 8 and 20 weeks post-ID BCG vaccination (red, *n* = 7–8) or MTBVAC vaccination (cyan, *n* = 6–8). *p < 0.05, **p < 0.01. Bars represent the median and IQR.

**Fig. 3:**
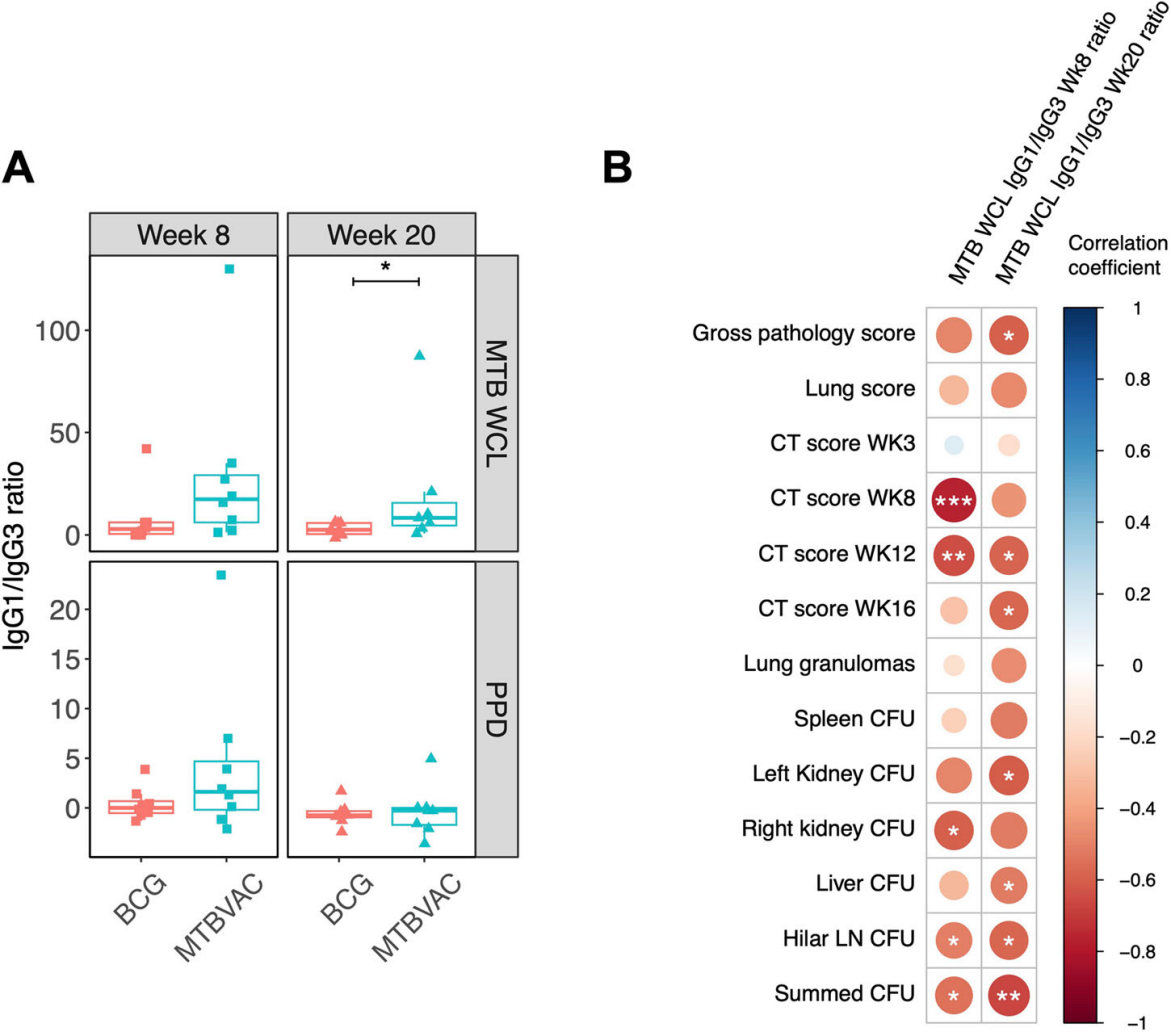
*M.tb*-specific IgG1/IgG3 ratio following BCG or MTBVAC vaccination. (**A**) *M.tb* WCL- and PPD-specific IgG1/IgG3 ratio in the sera of rhesus macaques collected at 8 and 20 weeks post-ID BCG vaccination (red, *n* = 8) or MTBVAC vaccination (cyan, *n* = 7–8). Bars represent the median and IQR. (**B**) Spearman’s correlations between *M.tb* WCL-specific IgG1/IgG3 ratio in the sera of rhesus macaques collected at 8 and 20 weeks post-vaccination with BCG or MTBVAC and measures of pathology following *M.tb* challenge, where the colour represents the correlation coefficient on a scale of −1 (dark red) indicating a negative correlation to 1 (dark blue) indicating a positive correlation. *p < 0.05, **p < 0.01, ***p < 0.001.

IgG subclass responses were then evaluated in serum from the same animals. While responses were modest, there was a significant increase in *M.tb* WCL- and PPD-specific IgG1 at 8 weeks post-vaccination in the MTBVAC group (*p* = 0.02 and *p* = 0.002 respectively), but altered responses were not detected in other subclasses in either group (Fig. 2A–H). MTBVAC-vaccinated animals had a significantly higher *M.tb* WCL-specific IgG1/IgG3 ratio at 20 weeks post-vaccination compared with BCG (*p* = 0.03, Fig. 3A).

When both groups were combined, there was a significant inverse correlation between *M.tb* WCL-specific IgG1/IgG3 ratio at week 8 post-vaccination and the CT scores at weeks 8 and 12 post-*M.tb* challenge (*r* = −0.76, *p* = 0.0006 and *r* = −0.65, *p* = 0.007 respectively); as well as CFU counts for the right kidney and hilar lymph nodes, and the summed CFU (*r* = −0.60, *p* = 0.015; *r* = −0.51, *p* = 0.044 and *r* = −0.54, *p* = 0.029 respectively). The *M.tb* WCL-specific IgG1/IgG3 ratio at week 20 post-vaccination was significantly inversely associated with the gross pathology score and CT scores at weeks 12 and 16 post-*M.tb* challenge (*r* = −0.60, *p* = 0.019; *r* = −0.58, *p* = 0.023 and *r* = −0.58, *p* = 0.031 respectively); as well as CFU counts for the left kidney, liver, hilar lymph nodes and the summed CFU (*r* = −0.60, *p* = 0.017; *r* = −0.52, *p* = 0.048; *r* = −0.58, *p* = 0.024 and *r* = −0.66, *p* = 0.007 respectively) (Fig. 3B). However, when each vaccine group was analysed alone, these associations did not hold apart from in the MTBVAC vaccinated group in which IgG1/IgG3 ratio at week 20 inversely correlated with the left kidney CFU (*r* = −0.76, *p* = 0.049). There were no associations between the PPD-specific IgG1/IgG3 ratio and measures of pathology following *M.tb* challenge.

### MTBVAC induces higher avidity *M.tb*-specific IgG compared with BCG that correlates with protection following *M.tb* challenge

**Fig. 4:**
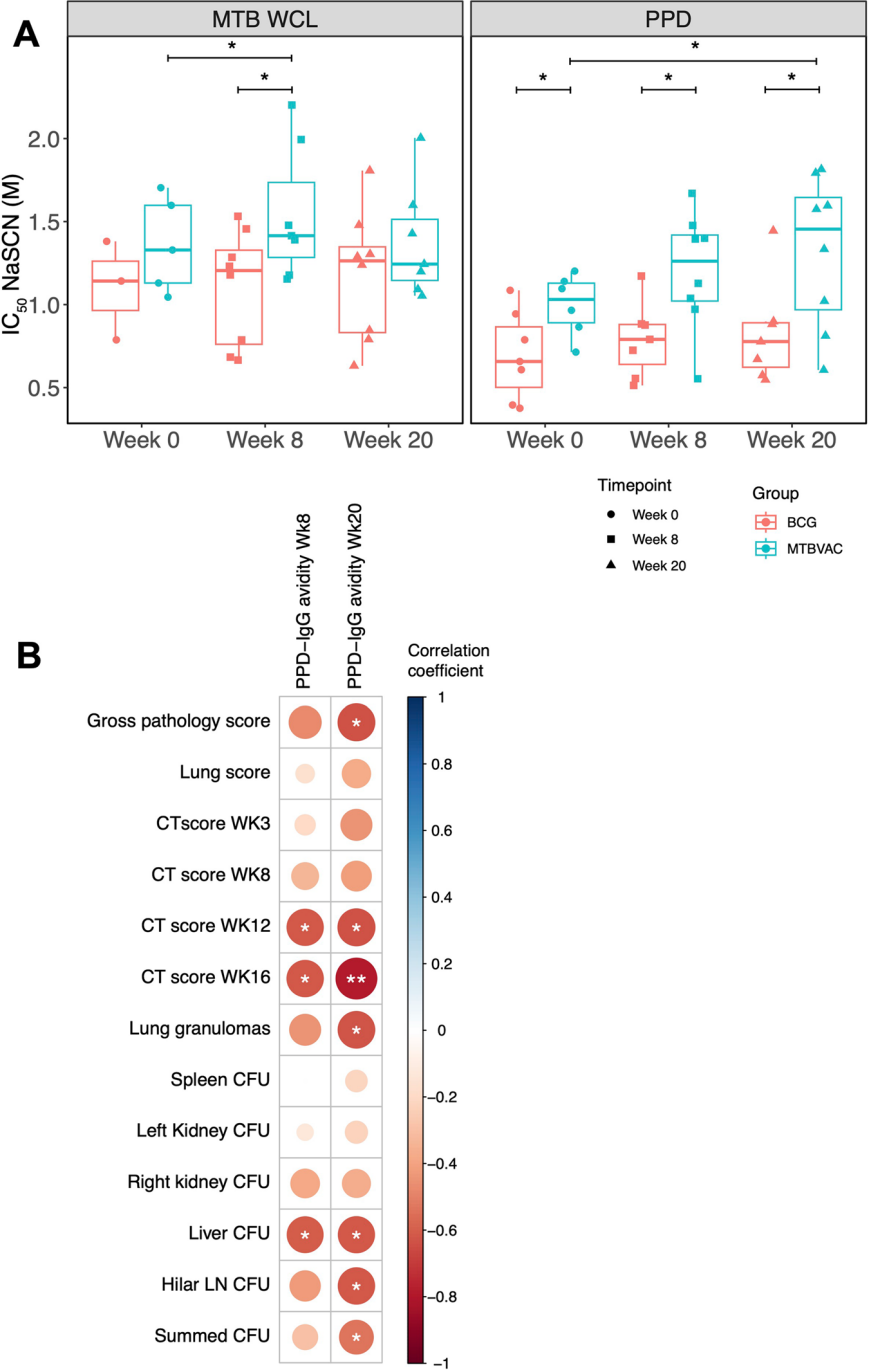
*M.tb*-specific IgG avidity following BCG or MTBVAC vaccination. **A** Avidity of *M.tb* WCL- and PPD-specific IgG in the sera of rhesus macaques collected at baseline and at 8 and 20 weeks post-ID BCG vaccination (red, *n* = 3–8) or MTBVAC vaccination (cyan, *n* = 5–8). Bars represent the median and IQR. **B** Spearman’s correlations between PPD-specific IgG avidity in the sera of rhesus macaques collected at 8 and 20 weeks post-vaccination with BCG or MTBVAC and measures of pathology following *M.tb* challenge, where the colour represents the correlation coefficient on a scale of −1 (dark red) indicating a negative correlation to 1 (dark blue) indicating a positive correlation. *p < 0.05, **p < 0.01.

Avidity of *M.tb*-specific IgG in serum from NHPs vaccinated with either BCG or MTBVAC by the ID route was evaluated at baseline and at 8 and 20 weeks post-vaccination. When comparing post-vaccination responses between groups, MTBVAC vaccinated animals demonstrated a superior response compared with ID BCG vaccinated animals at week 8 for *M.tb* WCL-specific IgG (*p* = 0.04, Fig. 4A, left panel) and at both weeks 8 and 20 for PPD-specific IgG (*p* = 0.03 and *p* = 0.04, Fig. 4A, right panel). However, there was also a difference between the groups in PPD-specific IgG avidity at baseline with animals in the MTBVAC group showing a significantly higher response (*p* = 0.04, Fig. 4B). Animals that received MTBVAC vaccination showed a significant increase in PPD-specific IgG avidity at week 20 (*p* = 0.04), and in *M.tb* WCL-specific IgG at week 8 (*p* = 0.02) compared to baseline. Avidity did not change following BCG vaccination compared to baseline.

When both groups were combined, PPD-specific IgG avidity at week 8 post-vaccination inversely correlated with CT scores at weeks 12 and 16 post-*M.tb* challenge (*r* = −0.61, *p* = 0.015 and *r* = −0.62, *p* = 0.02 respectively); as well as CFU counts in the liver (*r* = −0.60, *p* = 0.017). IgG avidity at week 20 inversely correlated with gross pathology score, CT scores at weeks 12 and 16, and lung granulomas (*r* = −0.64, *p* = 0.01; *r* = −0.78, *p* = 0.001 and *r* = −0.63, *p* = 0.01 respectively), as well as CFU counts in the liver and hilar lymph nodes and the summed CFU (*r* = −0.61, *p* = 0.02; *r* = −0.62, *p* = 0.01 and *r* = −0.53, *p* = 0.04 respectively) (Fig. 4B). Associations were not seen between avidity and pathology for the BCG group alone, but for the MTBVAC vaccinated group alone, there was a significant inverse correlation between IgG avidity at week 20 and the CT score at week 16 (*r* = −0.61, *p* = 0.02). Furthermore, fold change in IgG avidity at week 20 relative to baseline in this group was significantly inversely associated with the gross pathology score and the CT scores at weeks 3 and 16 (*r* = −0.89, *p* = 0.02; *r* = −0.93, *p* = 0.008 and *r* = −0.93, *p* = 0.008 respectively). All of the characteristics of the antibody responses measured in BCG and MTBVAC vaccinated animals are summarized in Supplementary Fig. 4.

## Discussion

We set out to better characterize the antibody response to MTBVAC vaccination and identify potential humoral immune correlates of its superior protective efficacy compared with BCG. In our study, MTBVAC vaccination, and to a lesser extent BCG vaccination, was capable of inducing increased *M.tb*-specific IgG responses in adult NHPs at 8 weeks post-vaccination (Fig. 1A,D). Unlike BCG, MTBVAC also stimulated increased *M.tb*-specific IgM and IgA titre (Fig. 1C,E,F). As IgA-producing plasma cells are mainly located at mucosal sites, this suggests that MTBVAC may be capable of inducing a mucosal immune response which could be beneficial in protecting against infection with *M.tb* which primarily invades the host through the mucosal surfaces of the respiratory tract.

It has previously been shown that respiratory or pulmonary vaccination with MTBVAC can induce functionally-relevant mucosal antibodies in animal models^28,29^. While systemic immunization has generally been considered incapable of eliciting such mucosal responses, recent studies suggest that other intradermally administered vaccines including the smallpox vaccine, a commercial inactivated *Mycoplasma hyopneumoniae* whole-cell vaccine and an inactivated influenza virus vaccine can induce the production of mucosal antibodies^30^. If samples had been available, it would have been interesting to quantify IgA responses in the bronchoalveolar lavage (BAL) of these animals; indeed Dijkman et al. previously noted a modest increase in *M.tb*-specific antibody levels in the BAL fluid of some NHPs that were vaccinated with MTBVAC by the ID route, although such mucosal responses were significantly enhanced in animals vaccinated by the pulmonary route^31^.

Avidity is a measure of the total strength of antibody binding across sites (as opposed to affinity which measures the strength of binding at any given individual site to an epitope)^32^. Antigen-driven selection of higher-affinity antibodies enhances the magnitude and longevity of host immunity through avidity maturation, which results from antigen presentation at germinal centres to promote fine-tuning of antibody complementarity^33^. Antibody avidity is a surrogate of protective efficacy for several vaccines against bacterial pathogens including meningococcus, pneumococcus, and *Haemophilus influenzae*^34–38^. Lu et al. recently described enhanced antibody avidity in ‘resisters’ (individuals who were highly exposed to *M.tb* but tested negative by IFN-γ release assay and tuberculin skin test, ‘resisting’ development of classic latent TB infection) compared to subjects with classic latent TB^39^. However, the effects of TB vaccines on antibody avidity have not been previously explored.

We demonstrate that the avidity of *M.tb*-specific IgG induced by MTBVAC vaccination is significantly greater than that induced by BCG at both 8 and 20 weeks post-vaccination. Although this may in part be driven by higher baseline responses in the MTBVAC group, there remained a significant increase between baseline and post-vaccination in the MTBVAC animals that was not observed in the BCG vaccinated animals (Fig. 4A). Notably, we observed significant inverse correlations between *M.tb*-specific IgG avidity post-vaccination and several measures of pathology following *M.tb* challenge in the same animals including CT scores, gross pathology score and granuloma count as well as CFU counts in various organs; some of which remained significant in the MTBVAC group alone indicating that the association is not merely an artefact of the two groups clustering for each measure (Fig. 4B). Importantly, the potential for IgG avidity to represent a functionally-relevant marker for TB vaccine-mediated protection is consistent with our other recent observation that BCG vaccination by the intravenous (IV) route, which confers substantially improved efficacy compared with standard ID BCG in NHPs^40,41^, induced an increase in *M.tb*-specific IgG avidity while ID BCG did not. Furthermore, IgG avidity correlated with ex vivo control of mycobacterial growth and protection following *M.tb* challenge in that study. Therefore, we have validated this association across two different independent TB vaccine strategies that confer superior protection compared with standard BCG. The *M.tb* challenge model used here investigates relatively early events occurring during infection and disease progression. It is important to determine whether the association with antibody avidity is dependent on disease phase, and whether it holds up in a reactivation model.

It was interesting to note that the frequency of PPD-specific IFN-γ producing cells following vaccination did not correlate with measures of pathology following *M.tb* challenge (Supplementary Fig. 2A). A central role for IFN-γ in the immune response to TB is undisputed^42–44^, and we have previously shown a reduced risk of TB disease in South African infants with higher levels of BCG-specific T cells secreting IFN-γ^25^. However, there are several reports showing a failure of IFN-γ responses to correlate with protection^45,46^. Furthermore, Lu et al. noted in the aforementioned study that although ‘resisters’ have the capacity to make IFN-γ in response to control antigens, they generate a non-IFN-γ-centric, *Mtb*-specific, CD4^+^ T cell response, marked by high levels of CD40L/CD154 upregulation, which may be key to the induction of *Mtb*-specific humoral immunity^39^.

To better understand the relationship between cellular immunity and antibody responses, we explored associations between PPD-specific IFN-γ producing cell frequency and PPD-specific antibody titre following vaccination. There were no significant correlations other than at week 16, when ELISpot responses were associated with measures of all isotypes at both timepoints studied (Supplementary Fig. 2B). In the case of the week 20 antibody response, this is likely the result of the kinetic of Th1 cell ‘help’ to B cells via signals that promote survival, proliferation, plasma cell differentiation, hypermutation, class-switch recombination, adhesion and chemoattraction^47^; it would be of interest to demonstrate such a mechanistic link, as independent induction remains a possibility. It is unclear why such a relationship would also be present at week 8, but this suggests that the early antibody response may be a predictor of the longer-term cellular memory response.

ESAT-6 and CFP-10 are major immunogenic proteins that are present in MTBVAC but not BCG; the latter having deletion of the RD1 region^27^. As noted, White et al. reported an increase in CFP-10- but not ESAT-6-specific IFN-γ ELISpot responses following MTBVAC vaccination that was significantly higher than that following BCG vaccination^20^. This was consistent with the finding of low-level, but significant, increases in CFP-10-specific IFN-γ-secreting cells in some MTBVAC-vaccinated human volunteers^21^. Although we did not see any overall differences in IgG responses to either antigen, two animals showed an increase in CFP-10-specific IgG at 20 weeks post-MTBVAC vaccination (Supplementary Fig. 3). Notably, CFP-10-specific IgG responses inversely correlated with gross pathology score following *M.tb* challenge in this group. By comparing the protection induced by BCG and MTBVAC in several mouse strains that naturally express different MHC haplotypes differentially recognizing ESAT-6 and CFP-10, Aguilo et al. previously linked reactogenicity to these antigens to improved protection against *M.tb*^48^. Our findings provide support for this association in NHP, with the caveat that we observed low numbers of CFP-10 responders, necessitating larger studies to validate the finding in this model.

In humans, a Th1 response is generally considered to be associated with IgG1 and IgG3 production, while Th2 responses are linked to the generation of IgG4^49^. In TB, where the specific pattern of cytokines is predominantly Th1, IgG1 is the most prevalent subclass for all forms of disease, where it has been shown to be part of the pro-inflammatory response and to stimulate the release of TNF-α production from primary monocytes^50,51^. Less is known about IgG subclasses and progression of subclass switching in macaques, although Bjornson-Hooper et al. have mapped the species-specific immunophenotype and cytokine profile to conventional stimuli in mice, NHPs and humans, with comparative analysis of signalling responses in T and B cells showing broad similarities between NHPs and humans with important roles for the amplification of the immune response and production of antibodies^52^. As in humans, IgG1 has been reported to be the most functional subclass and appears to be associated with stronger Th1 responses in vaccine studies^53–56^. Although titres were generally low in our subclass analysis, IgG1 clearly predominated, with significant increases in *M.tb*-specific IgG1 following MTBVAC but not BCG vaccination (Fig. 2).

We have previously reported that IgG1 responses to specific *M.tb* antigens correlate with improved control of mycobacterial growth in an ex vivo mycobacterial growth inhibition assay^57^, suggesting a role for this subclass in protection against TB. Furthermore, passive immunization with a monoclonal antibody (SMITB14) of the IgG1 subclass to lipoarabinomannan and its corresponding F(ab’) have been shown to confer protection against *M.tb* challenge in mice^58^. In other disease models, including COVID-19 and malaria, the relative amounts of IgG1 and IgG3 (or the IgG1:IgG3 ratio) have been associated with enhanced neutralizing capacity and reduced risk^59,60^. To our knowledge, the IgG1:IgG3 ratio has not been previously explored in the context of TB. Although we detected very low level responses, our finding of an increased ratio in MTBVAC compared with BCG vaccinated animals and an inverse association with pathology following *M.tb* challenge (Fig. 3) suggests this measure may warrant further exploration.

This study harbours some limitations. The small sample size of eight animals per group may limit the sensitivity to detect differences between groups and associations with measures of protection. The administered dose of MTBVAC was also lower than that of BCG, which might result in underestimates of superiority in the comparative analysis. Additionally, due to limiting sample volume the study did not define specific antigens which may have been responsible for the differences in antibody response between the two vaccine groups, or incorporate functional assays, which could have provided mechanistic insights and further supported putative correlates of protection. While these results are informative, it is critical to recognize that they are based on an NHP model of vaccination and infection. Therefore, it is essential to confirm these findings in humans and further explore these responses as potential biomarkers of protection in MTBVAC clinical trials.

## Methods

### Non-human primate studies

Stored serum samples collected at baseline and at 8 and 20 weeks post-vaccination with BCG or MTBVAC were used from a historical NHP study to avoid the use of additional animals. MTBVAC vaccination conferred superior protection against aerosol *M.tb* challenge compared to BCG vaccination in that study, offering a valuable opportunity for immune correlates investigations^20^.

#### Experimental animals

Serum samples were previously collected from 16 male and female Rhesus macaques of Indian genotype from an established closed UK breeding colony (*n* = 4 males and *n* = 4 females in each experimental group). Animals were aged between 4.2 and 5.2 years and each individual animal was considered an experimental unit (as defined by the ARRIVE guidelines as “the biological entity subjected to an intervention independently of all other units, such that it is possible to assign any two experimental units to different treatment groups”). None of the animals had been used previously for experimental procedures, and were housed in socially compatible groups of four. Each socially compatible housing group was randomly allocated to a study treatment using computer-generated random assignment: to receive vaccination with either BCG (*n* = 8) or MTBVAC (*n* = 8). The sample size per group was previously determined as the minimum required to detect a nine-point reduction in total pathology score as the primary efficacy readout, with a power of 80% and an α of 0.05^20^. No animals were excluded during the experiment. Not all serology analyses could be run on all samples due to serum volume limitations; this is the only reason for incomplete datasets.

Animals were housed in compatible social groups, in accordance with the Home Office (UK) Code of Practice for the Housing and Care of Animals Used in Scientific Procedures (1989), and the National Committee for Refinement, Reduction and Replacement (NC3Rs), Guidelines on Primate Accommodation, Care and Use, August 2006 (NC3Rs, 2006). Animal procedures and study designs were approved by the Establishment of Animal Welfare and Ethical Review Committee and authorised under a UK Home Office project licence. Absence of previous exposure to mycobacterial antigens was confirmed by ex vivo IFN-γ ELISpot (MabTech, Nacka. Sweden) to measure responses to mycobacterial antigens: PPD (SSI, Copenhagen, Denmark), and pooled 15-mer peptides of ESAT-6 and CFP-10 (Peptide Protein Research Ltd, Fareham, UK). Animals were sedated by intramuscular injection of ketamine hydrochloride (Ketaset, 100 mg/ml, Fort Dodge Animal Health Ltd, Southampton, UK; 10 mg/kg) for procedures requiring removal from their housing.

#### BCG and MTBVAC vaccinations

BCG vaccinations were performed using BCG Danish strain 1331 (1st WHO reference reagent, NIBSC, UK), prepared according to the manufacturer’s instructions by addition of 1 ml Sauton’s diluent to a lyophilized vial to give an estimated concentration of 2–8 × 10^6^ CFU/ml. To prepare MTBVAC, 1 ml of sterile water was added to a lyophilized vial to give a concentration of 3–17 × 10^6^ CFU. Culture of residual vaccine on solid agar confirmed the viability of the vaccines, and the mean average administered dose of each vaccine was calculated to be 1.2 × 10^6^ (SD 3.4 × 10^4^) CFU and 8.2 × 10^5^ (SD 3.9 × 10^4^) CFU for BCG and MTBVAC respectively. Vaccines were administered in a volume of 100 µl intradermally (ID) into the upper left arm under sedation. BCG- and MTBVAC-vaccinated animals were housed separately. The injection sites were monitored for local reactions after vaccination, and any reactions were measured and assessed. Mild induration and erythema occurred at the site of immunisation in all animals that received an ID vaccination with BCG and in five of the eight animals that received ID vaccination with MTBVAC. The skin reactions induced were comparable in size and resolved within six weeks after MTBVAC vaccination and between six and 14 weeks after BCG.

#### *M.tb* challenge and measures of disease burden

All animals were experimentally infected with *M.tb* Erdman strain K01 (HPA-Sept 2011 stock, prepared from stocks originating from BEI Resources) via the natural aerosol route at 21 weeks after vaccination. Mono-dispersed bacteria in particles were generated using a 3-jet collison nebuliser (BGI) and, in conjunction with a modified Henderson apparatus, delivered to the nares of each sedated animal via a modified veterinary anaesthesia mask. Challenge was performed on sedated animals placed within a ‘head-out’, plethysmography chamber (Buxco, Wilmington, North Carolina, USA). The retained dose was estimated to be approximately five viable CFU, calculated as previously described^20,61,62^. Clinical examinations were performed under sedation every two weeks throughout the study, including thoracic radiographs, measurement of body weight and temperature, and collection of blood samples to determine haemoglobin levels and ESR. Behaviour was also monitored for contra-indicators. All animals showed the weight gain profiles expected in normal healthy animals during the period prior to challenge and were unperturbed by vaccination. Body temperature, erythrocyte sedimentation rate (ESR) and red cell haemoglobin concentration remained within the normal range for the species during the period between vaccination and challenge in all animals.

Computed tomography (CT) scans were performed on sedated animals using a 16-slice Lightspeed CT scanner (General Electric Healthcare, Milwaukee, WI, USA) at 3, 8, 12 and 16 weeks after *M.tb* challenge, as previously described^20,63^. Niopam 300 contrast medium (Bracco, Milan, Italy) was administered intravenously at 2 ml/kg body weight to allow full examination of lesions and lymph nodes. An expert thoracic radiologist blinded to treatment group and clinical status evaluated scans as previously described^20^, and the disease burden attributable to *M.tb* infection was scored using a relative scoring system based on the number of lesions present in lungs, spleen, liver, kidney and lymph nodes, and the presence and extent of TB-induced structural abnormalities^64^. The scores for each tissue (lung lobe, organ, lymph node) were summed to give a total CT score at each timepoint, providing a combined measure of pulmonary and extrapulmonary disease burden.

Necropsy was performed by intracardiac injection of a lethal dose of anaesthetic (Dolelethal, Vétoquinol UK Ltd, 140 mg/kg) at 16–18 weeks after *M.tb* challenge or when the disease progressed to meet the predefined human endpoint criteria. If prior to the end of the planned study period, time of necropsy was determined by experienced primatology staff and based on a combination of the following adverse indicators: depression or withdrawn behaviour, abnormal respiration (dyspnoea), loss of 20% of peak post-challenge body weight, ESR levels elevated above normal ( > 20 mm), haemoglobin level below normal limits ( < 100 g/dL), increased temperature ( > 41 °C) and abnormal findings on the thoracic radiographs. A post-mortem examination was performed immediately following necropsy, and pathological changes were scored using an established system based on the number and extent of lesions present in the lungs, spleen, liver, kidney and lymph nodes, as previously described^20,65^. All gross and histopathological examinations were carried out by a qualified veterinary pathologist blinded to the treatment group. Samples of spleen, liver, kidneys and tracheobronchial lymph nodes were removed and sampled for the presence of viable *M.tb* by homogenizing weighed tissue samples in sterile water, followed by plating onto Middlebrook 7H11 OADC selective agar. Plates were incubated for three weeks at 37 °C before quantification of *M.tb* colonies^20^.

### Standardised indirect enzyme-linked immunosorbent assays (ELISAs)

96-well NUNC MaxiSorp plates from Thermo Fisher (high protein binding) were coated with 50 µL of antigen solution diluted in Dulbecco’s Phosphate Buffered Saline (DPBS) and incubated for 16 hr to 18 hr at 4 °C. The antigens used for coating were *M.tb* whole cell lysate (WCL) (1 µg/ml) and *M.tb* purified protein derivative (PPD) (5 µg/ml) (AJ Vaccines, Denmark). Plates were then blocked with 100 µl of casein-PBS blocker solution for 1 hr and incubated for 2 hr with 50 µl of NHP serum diluted 1:100 in casein. Plates were then incubated for 1 hr with 50 µl/well of a 1:1000 dilution in casein of either goat anti-monkey IgG γ-chain-specific, goat anti-monkey IgM μ-chain-specific, goat anti-monkey IgA α-chain-specific, anti-monkey IgG_1_ γ-chain-specific, anti-monkey IgG_2_ γ-chain-specific, anti-monkey IgG_3_ γ-chain-specific, or anti-monkey IgG_4_ γ-chain-specific secondary antibodies conjugated to either alkaline phosphatase (AP) or biotin (Rockland Laboratories). In the case of biotin-conjugation, 50 µl of 1:500 of Extravidin®-AP (Sigma-Aldrich, MA) was added and incubated at room temperature for 1 h. Finally, 100 µl of 4-Nitrophenyl phosphate disodium salt hexahydrate (pNPP) developing solution was added, and plates were subsequently read every 30 min.

The standard curve used on each plate was derived from a pool of NHP sera previously established to contain a high-titre of antibodies against the antigen mixture being tested. A 1:100 dilution of the standard pool was used in a two-fold serial dilution series to produce ten standard points that were assigned arbitrary ELISA units. The optical density values of the standard points were fitted to a four-parameter hyperbolic curve against the arbitrary ELISA units using Gen5 software (version 3.04; BioTek Instruments, Winooski, VT, USA), and the parameters estimated from the standard curve were used to convert absorbance values of individual test samples into ELISA units. Each ELISA plate consisted of the samples and internal positive control (1:800 dilution of the standard pool, corresponding to standard 4) in triplicate, ten standard points in duplicate, and four blank negative control wells. The optical density reading of the plates at 405 nm was performed using an Elx808 microplate reader (BioTek Instruments). Plates were washed 4x in PBS-Tween and tapped dry between each step, except after blocking where washing was not performed.

### Avidity assay

96-well NUNC MaxiSorp plates from Thermo Fisher (high protein binding) were coated with 50 µl of antigen solution diluted in DPBS and incubated for 16 h to 18 h at 4°C. The antigens used for coating were either *M.tb* WCL (1 µg/ml) or PPD (5 µg/ml). Plates were then blocked with 100 µl of casein-PBS blocker solution for 1 h and then incubated for 2 h with 50 µl of serum diluted 1:100 in casein. 50 µl of the chaotropic agent sodium thiocyanate (NaSCN) was added in increasing concentrations to the samples and incubated for 15 minutes at room temperature. In all cases NaSCN concentrations ranged from 0 M to 4 M. 100 µl of pNPP developing solution was added. Plates were then incubated for 1 h with 50 µl/well of 1:1000 dilution in casein of goat anti-monkey IgG γ-chain-specific secondary antibody conjugated to AP (Rockland Laboratories). In the case of the *M.tb* WCL experiments, plates reached an initial OD of 1.0 after approximately 1 h; in the case of PPD it was after 30 minutes. The reading was performed at a wavelength of 405 nm with a BioTek2 ELISA plate reader. Plates were washed 4× in PBS-Tween and tapped dry between each step, except after blocking. For each sample data the response was normalised from 0 to 100% using GraphPad Prism software. Then, the IC50 for each sample was calculated.

### Statistical analysis

Data were analysed using GraphPad Prism v.9.2 and SPSS v.29.0. Non-parametric tests were employed due to small sample sizes. Longitudinal data was analysed using a Friedman test with Dunn’s correction for multiple comparisons (all time-points vs. baseline). For studies with random missing values due to sample unavailability, data was logged and analysed using a mixed-effects model with Dunnett’s correction for multiple comparisons (all time-points vs. baseline). When comparing two conditions, a Wilcoxon signed-rank test was conducted; where there were random missing values due to sample unavailability, unpaired data was excluded, or if there were multiple pairs each with one missing value, an unpaired Mann-Whitney test was used. Associations were determined using a two-tailed Spearman’s rank correlation test. Figures were plotted using R version 4.0.1.

## Supplementary information

Supplementary Information

## Data Availability

The data included in this study are available upon reasonable request.
