# Supplementary material for: MTBVAC induces superior antibody titers and IgG avidity compared to BCG vaccination in non-human primates
Source: NPJ Vaccines. 2024 Nov 20;9:230. doi: 10.1038/s41541-024-01009-5 (PMC11579480; doi:10.1038/s41541-024-01009-5)
Supplement: Supplementary file 1 — Supplementary Information [file 41541_2024_1009_MOESM1_ESM.pdf]

MTBVAC vaccination induces superior antibody titers and enhanced IgG avidity that is associated with superior protection from *M.tb* challenge in non-human primates compared with BCG vaccination

__Supplementary information__

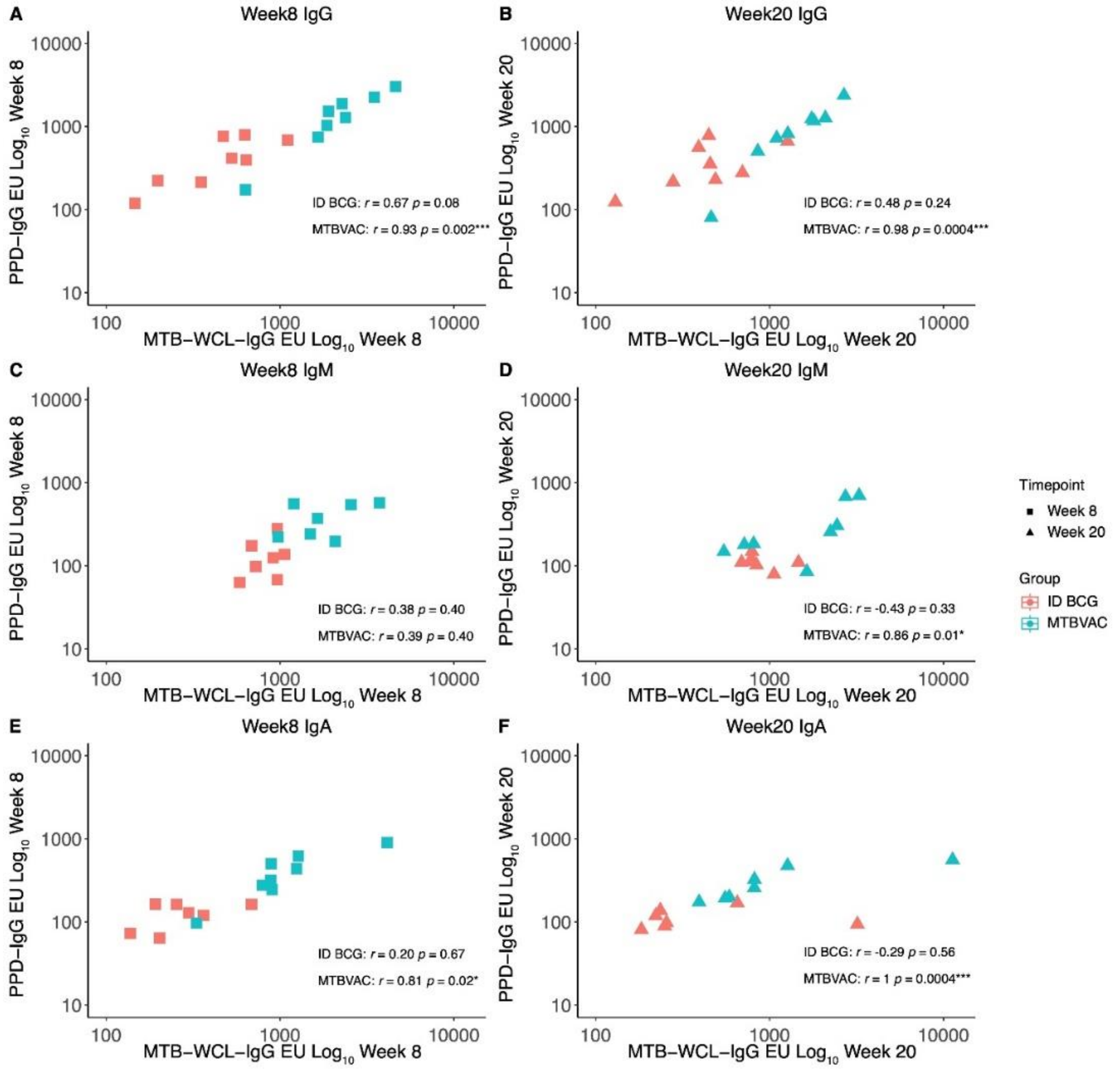

Supplementary Figure 1. Spearman's correlations between IgG (A), IgM (C) and IgA (E) responses to *M.tb* WCL and PPD in the sera of rhesus macaques collected at 8 weeks post-ID BCG vaccination (red) or MTBVAC vaccination (cyan). IgG (B), IgM (D) and IgA (F) responses to *M.tb* WCL and PPD in the sera of rhesus macaques collected at 20 weeks post-ID BCG vaccination (red) or MTBVAC vaccination (cyan).

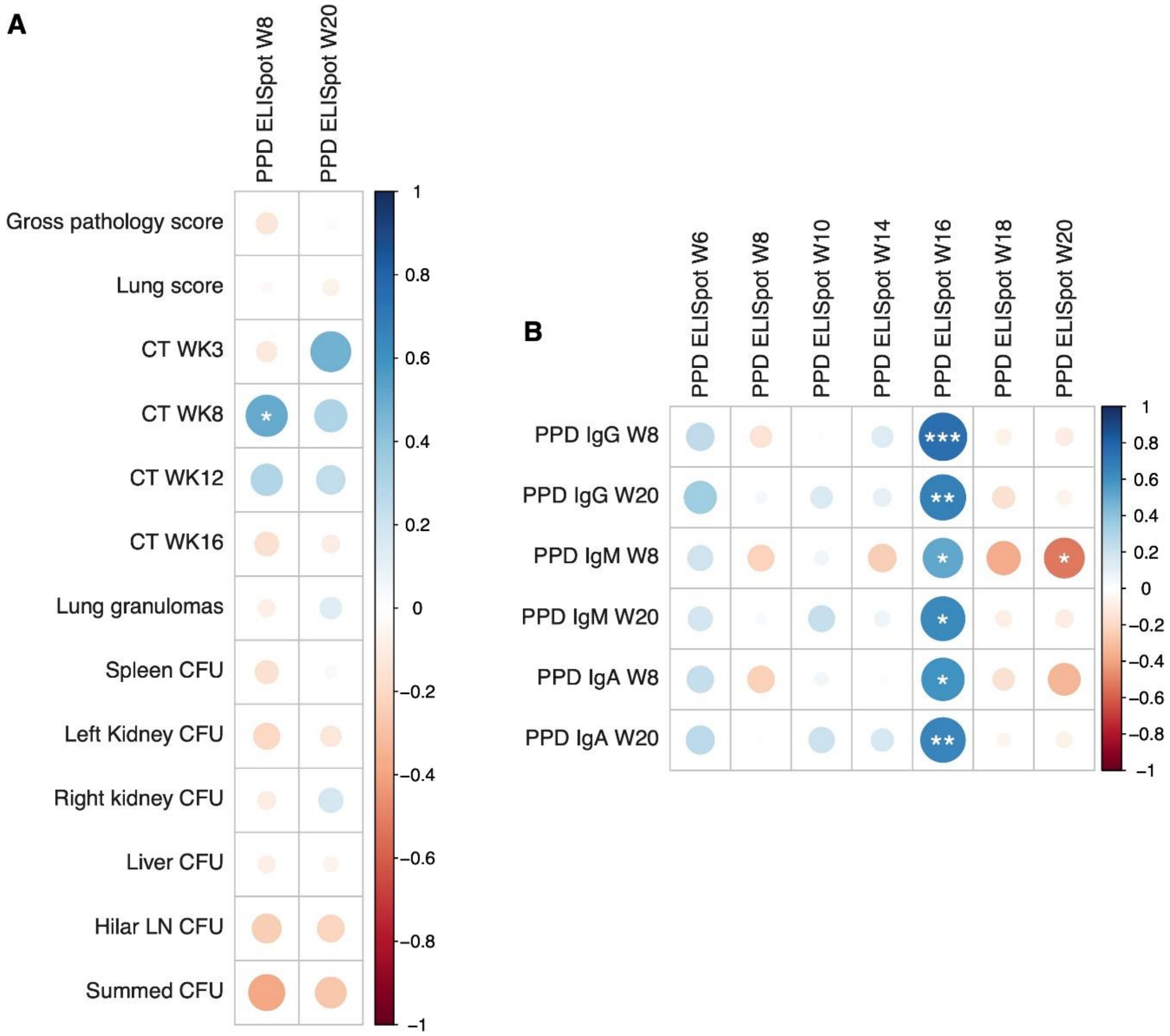

Supplementary Figure 2. Correlation matrices of Spearman's correlations between measures of pathology following *M.tb* challenge and PPD-specific ELISpot responses at 8 or 20 weeks post-vaccination (A), and between PPD-specific ELISpot responses and PPD-specific antibody responses post-vaccination (B). The colour represents the correlation coefficient on a scale of -1 (dark red) indicating a negative correlation to 1 (dark blue) indicating a positive correlation. * denotes p<0.05, ** denotes p<0.01, *** denotes p<0.001.

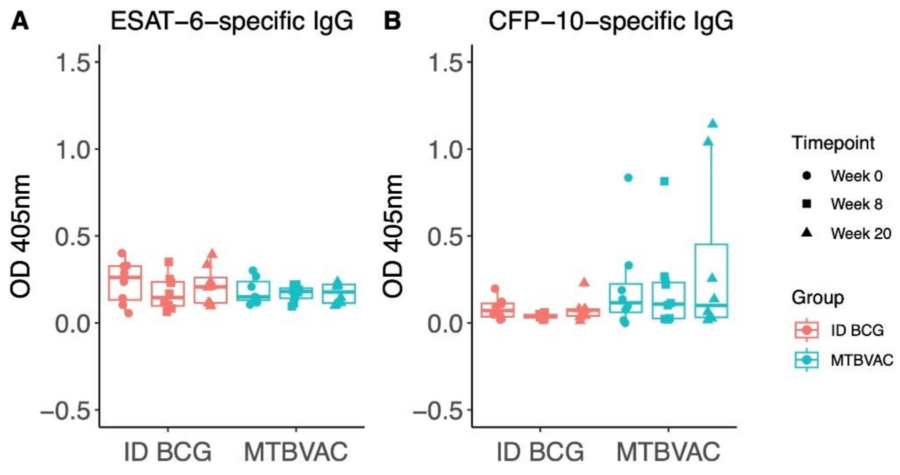

Supplementary Figure 3. IgG responses specific to ESAT6 (A) and CFP10 (B) in the sera of rhesus macaques collected at baseline, 8 and 20 weeks post-ID BCG vaccination (red) or MTBVAC vaccination (cyan).

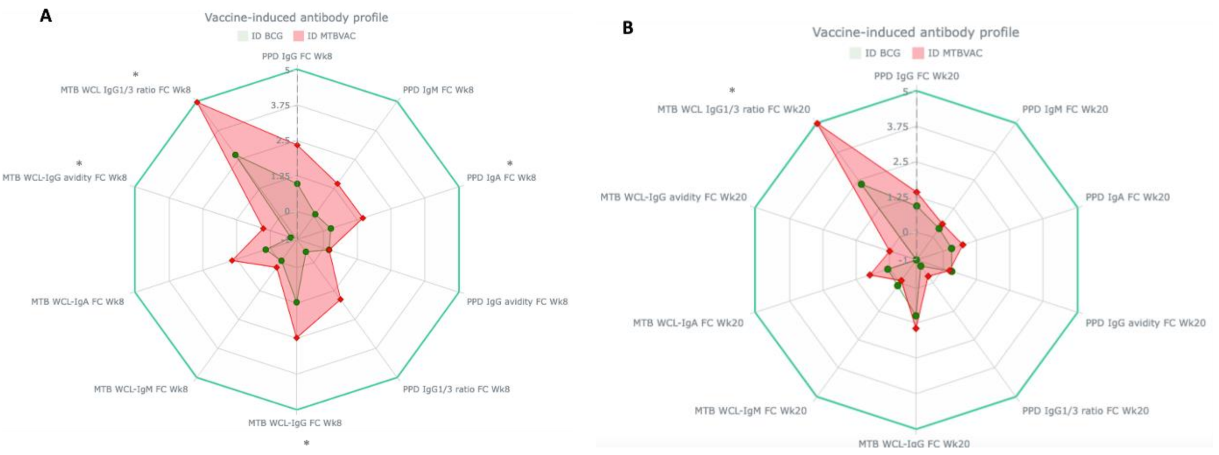

Supplementary Figure 4. Summary of the vaccine induced profiles at week 8 (A) and 20 (B) post-vaccination with BCG (green) or MTBVAC (red). Each spoke shows fold change in response relative to baseline of the isotype indicated to the antigen indicated at the timepoint indicated.
